# Correlation of Sperm Parameters With Semen Lipid Peroxidation and Total Antioxidants Levels in Astheno- and Oligoasheno- Teratospermic Men

**DOI:** 10.5812/ircmj.6409

**Published:** 2013-09-05

**Authors:** Abasalt Hosseinzadeh Colagar, Fatemeh Karimi, Seyed Gholam Ali Jorsaraei

**Affiliations:** 1 Department of Molecular and Cell Biology; Nano and Biotechnology Research Group, Faculty of Basic Sciences, University of Mazandaran, Babolsar, IR Iran; 2 Fatemeh-Zahra Infertility and Health Reproductive Research Center, Babol University of Medical Sciences, Babol, IR Iran

**Keywords:** Lipid Peroxidation, Malondialdehyde, Reactive Oxygen Species

## Abstract

**Background:**

Sperm dysfunction caused by reactive oxygen species (ROSs) is one of the major causes of infertility in men, which leads to, lipid peroxidation (LPO) and the formation of stable peroxidation products like Malondialdehyde (MDA) in seminal plasma. MDA is effective factor in reducing fertility.

**Objectives:**

The aim of this study is to determine two biochemical markers of oxidative stress; TAC and MDA, and them correlation to quality-quantity factors in Asthenoteratospermic and Oligoashenoteratospermic men.

**Patients and Methods:**

A total of 42 semen samples including: 15 samples normospermic as control group, 12 Asthenoteratospermic and 15 oligoasthenoteratospermic were collected from Babol IVF center; Iran. Semen analysis was performed according to WHO (1999) guidelines. Seminal plasma TAC and MDA levels in all patients were measured by TBARs and FRAP methods, respectively.

**Results:**

Seminal plasma TAC level in normospermic men was significantly higher than asthenoteratospermic men (P < 0.001) and oligasthenoteratospermic men (P < 0.001) and had posetive correlation with sperm count, motility and morphology. In contrast MDA levels in normospermic men were significantly lower than in asthenoteratospermic men (P = 0.049) and oligoasthenoteratospermic men (P = 0.001) and had negative correlation with sperm count, motility and morphology.

**Conclusions:**

These results suggest that lipid peroxidation and decreasing total antioxidant capacity lead to low motility; morphology and sperm count in spermatozoa of astheno-and oligoastheno-teratospermic men. Therefore, evaluation of oxidative status and antioxidant defenses system may be as a useful tool for diagnosis and treatment of male infertility especially in idiopathic male infertility.

## 1. Introduction

Oxidative stress is an important factor which influences fertility potential of spermatozoa by lipid peroxidation which may result in sperm dysfunction. Sperm count and sperm motility are fundamental parameters that ascertain the functional ability of spermatozoa (1). Decreased the sperm motility (asthenozoospermia) is considered to associate with the infertility of a significant number of males, and many cases of reduction in the sperm motility are not completely understood. Many factors can affect sperm motility, but they are still not clear. One of them that potentially causes asthenozoospermia is oxidative stress induced by ROS (2-4). The most common ROS that have potential significance in reproductive biology, include the superoxide anion(O_2_^-^), hydrogen peroxide (H_2_O_2_), the peroxyl (ROO^-^) and the hydroxyl (OH^-^) radicals (5, 6). Reactive oxygen species (ROS) has both physiological and pathological roles in male infertility. The physiological level of ROS plays a crucial role in processes such as maturation, capacitation, acrosomal reactions, and fertilization (7-10). On the other hand, pathological levels of ROS, which can originate from endogenous sources such as leukocytes (11, 12) and immature/abnormal spermatozoa (9, 12) or from exogenous sources such as environmental factors (e.g. cigarette smoking, alcohol) (13, 14) can be potentially toxic for spermatozoan function due to the peroxidation of high polyunsaturated fatty acids (PUFA) within the plasma membrane of spermatozoa (5, 11, 15) Increased ROS levels also have been associated with reduction in the sperm motility (16-18). However, the link between ROS and reduced motility in spermatozoa is not fully understood. Thus, many hypotheses have been proposed to explain it.

One hypothesis is that H_2_O_2_ can diffuse across the cell membrane into the cytoplasm and inhibit the activity of enzymes such as glucose-6-phosphate dehydrogenase (G6PD). This enzyme controls the rate of glucose flux via the hexose monophosphate shunt which in turn, controls the intracellular availability of nicotinamide adenine dinucleotide phosphate (NADPH). This in turn is used as a source of electrons by spermatozoa to fuel the generation of ROS by an enzyme system known as NADPH oxidase (19). Inhibition of G6PD leads to a decrease in the availability of NADPH and a concomitant accumulation of oxidized glutathione and reduced glutathione. This can reduce the antioxidant defenses system of the spermatozoa and increase membrane phospholipids peroxidation (20). Another hypothesis involves a series of cascade chemical reactions that result in a decrease in axonemal protein phosphorylation and reduce sperm motility, both of which are associated with a reduction in membrane fluidity and sperm-oocyte fusion (21). Malondialdehyde (MDA) is one of the reactive and mutagenic aldehyde products of lipid peroxidation in seminal plasma (22) Toxic lipid peroxides are known to cause different impairments of sperm cells and may play a main role in the etiology of male infertility. Malondialdehyde (MDA) is an indicator of lipid peroxidation which may be a diagnostic tool for the analysis of infertility (23, 24).

## 2. Objectives

We determined the correlation between the TAC and MDA concentration with the motility, morphology and sperm count of spermatozoa in asthenoteratospermic and oligoasthenoteratospermic in comparison with control men.

## 3. Patients and Methods

Forty-five semen samples from men in the age range 24 - 38 years attending the infertility clinic at Fatemeh Zahra Hospital (Babol, Iran) were collected into sterile containers after a period for 2-3 days of abstinence. Semen specimens were allowed to liquefy at 37°C for 30 min. Routine analysis of semen was performed within 1 hour according to World Health Organization guidelines (25). A hematoxylin-eosin (H&E) staining method was used for determination of the percent normal morphology of spermatozoa. Samples were then classified as “normozoospermic”, “asthenotratospermic” (with motility < 50% and morphology < 14%) and “oligoasthenoteratospermic“(with Sperm concentration < 20 million per ml, motility < 50% and morphology < 14%). Morphology of the spermatozoa was assessed using Kruger’s criteria that morphology < 14% is considered abnormal (26).

### 3.1. Measurement of TAC

Semen samples were centrifuged at 14000 ×g for 7 min. after centrifugation, supernatants were diluted 1:10 v/v in distilled water. TAC was then evaluated using ferric-reducing ability of plasma (FRAP) according to method of Benzie (1996) (27) with slightly modifications. This method shows the ability of seminal plasma antioxidants to reduce ferric-tripyridyltriazine (Fe^3+^ - TPTZ) to a ferrous form (Fe^2+^). For TAC measurement, the working FRAP reagent was prepared by mixing 10 vol. of 300 mmol/l acetate buffer; pH 3.6 with 1 vol. of 10 mmol/l 2, 4, 6,-tripyridyl-s-triazine in 40 mmol/l HCl with one volume of 20 mmol/l FeCl_3_.6H_2_O. Then, 1.5 ml of the working FRAP reagent was aliquoted into a glass tube and warmed to 37°C for 5 minutes. Subsequently, 50 μL of plasma and 50 μL of distilled water (reagent-free) as well as 50 μL of each of the standard solutions (FeSO_4_.7H_2_O; 1000, 500, 250, 125 μM) were added to 1.5 mL FRAP reagent and heated to 37°C for 10 minutes. Absorbance was measured at 593 nm using a spectrophotometer (UV-visible). The final results were expressed as μM/l.6.

### 3.2. Measurement of MDA

Seminal MDA levels were determined according to the method as described by Rao et al. (28), which was slightly modified by Gholinezhad and Hosseinzadeh Colagar (6).

### 3.3. Statistical Analysis

Data are presented as mean ± S.D. An independent t-test using SPSS 16 for Windows software (SPSS Incorporated Chicago, IL, USA) was considered to analyze data collected. A linear regression (Spearman) model was applied to the relationship of MDA levels and TAC with the motility and morphology of spermatozoa. In all cases, P < 0.05 was considered statistically significant.

## 4. Results

Semen analysis was done according to WHO guidelines 25.The concentration of MDA and the TAC in seminal plasma was measured by TBARs and FRAP methods, respectively.

### 4.1. Semen Analysis

The parameters investigated in semen samples are shown in Table 1. 

**Table 1.** Standard Semen Parameters Analysis in Healthy, Asthenoteratospermic and Oligoasthenoteratospermic Men

Semen Parameters ^a^	C ^b^	AT ^b^	OAT ^b^
**Volume (mL)**	3.067 ± 0.88	2.29 ± 1.01^e^	2.60 ± 0.92
**Sperm count (× 106/mL)**	96.67 ± 8.16	47.75 ± 22.97 ^c^	5.07 ± 2.4 ^c^
**Total sperm (× 106)**	292.33 ± 78.03	108.0 ± 66.68 ^c^	12.71 ± 6.98 ^c^
**Sperm motility, No. (%)**	62.66 ± 7.98	33.50 ± 4.66 ^c^	20.53 ± 14.54 ^c^
**Sperm morphology, No. (%)**	27.86 ± 9.34	7.58 ± 2.61 ^c^	6.5 ± 3.22 ^c^

^a^ Values expressed as mean ± SD. Semen parameter in patient groups compared with control group. P < 0.05 was considered statistically significant.

^b^ Abbreviations: C, Controls (n = 15); AT, asthenoteratospermic (n = 12); OAT, oligoasthenoteratospermic (n = 15)

^e^ P < 0.05

^c^ P < 0.001

**Figure 1.**
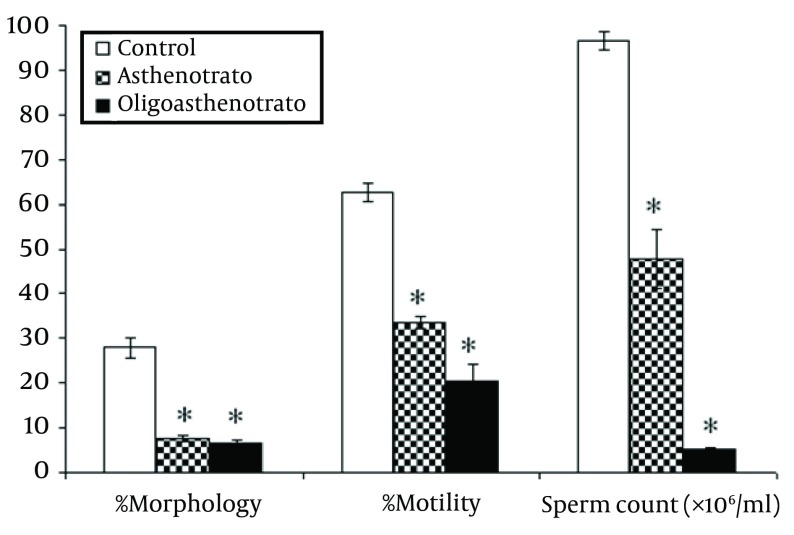
Comparison of Percentage of Motility, Morphology and Sperm Count (× 106/Ml) Between Control (C) and Patient Groups (AT, OAT) Percentage of normal sperm morphology between patient groups was significant (P = 0.006). Error bar, representing SEM. P < 0.05 was considered statistically significant, * P < 0.001

All parameters (count, total count, motility and morphology of spermatozoa) in control men were strongly significant (P < 0.001) in comparison with patient groups (Figure 1). 

Semen volume in asthenotratospermic was also significantly lower than in healthy men (P < 0.05 respectively) and in oligoasthenoteratospermic was lower than in healthy men (P = 0.13) but this relation was not significant.

### 4.2. TAC and MDA Assays

Comparison of the TAC and MDA levels are shown in Table 2. 

**Table 2.** Comparison of TAC and MDA Concentration in Healthy, Asthenoteratospermic and Oligoasthenoteratospermic Men ^a ^

				P value		
**Variables**	**C**	**AT**	**OAT**	**C vs. AT**	**C vs. OAT**	**AT vs. OAT**
**TAC (µM/l)**	3239 ± 562.25	1916.4 ± 575.39	1896.7 ± 650.86	< 0.001	< 0.001	0.103
**MDA(nmol/ml)**	0.569 ± 0.20	0.889 ± 0.25	0.932 ± 0.31	0.049	0.001	0.473

^a^ Values expressed as mean ± SD and P < 0.05 was considered statistically significant.

**Figure 2.**
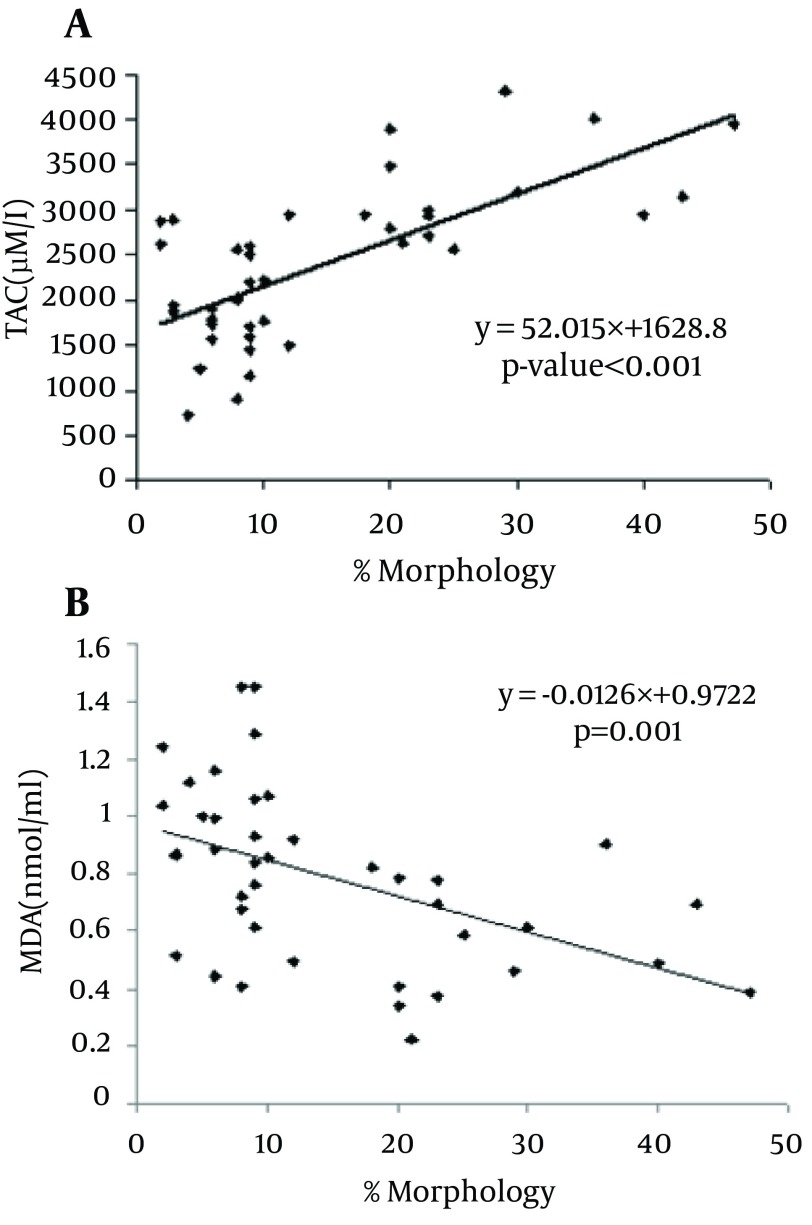
Correlation of Sperm Morphology With Total Antioxidant Capacity/TAC A) Malondialdehyde/MDA, B).Correlation is Significant at the P < 0.05 Level (2-Tailed).

**Figure 3.**
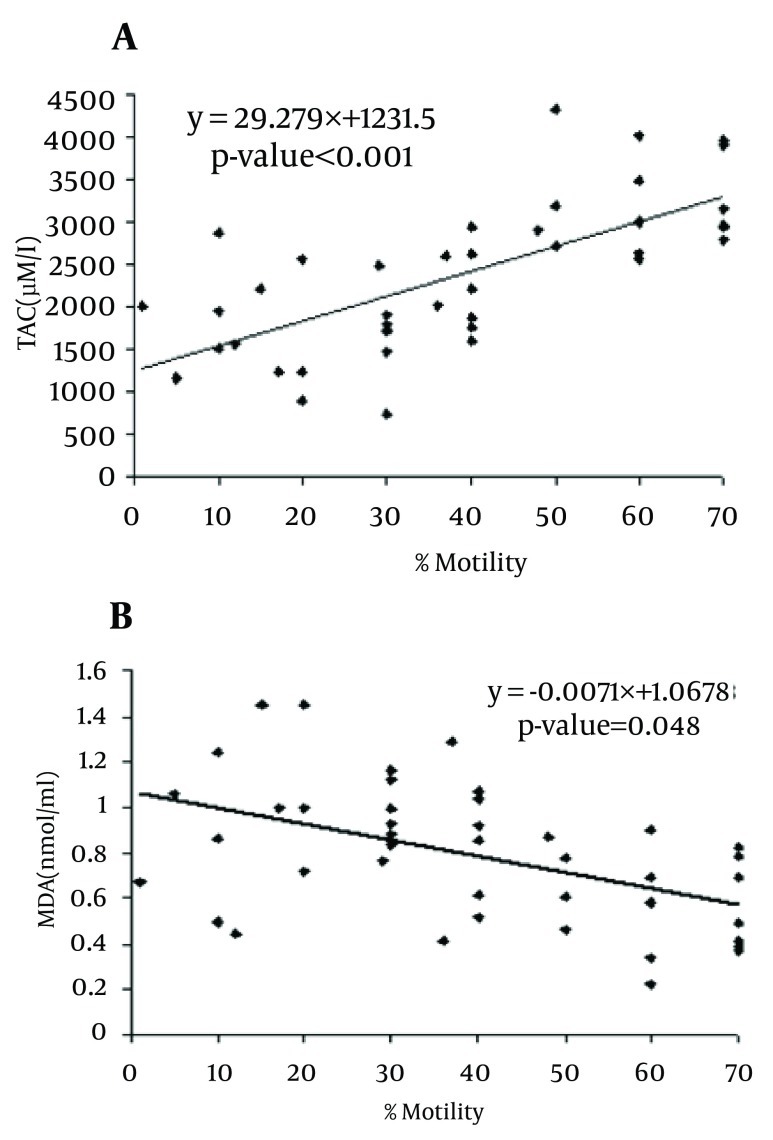
The Correlations of the Sperm Motility with TAC A) MDA, B) Correlation is significant at P < 0.05 level (2-tailed).

**Figure 4.**
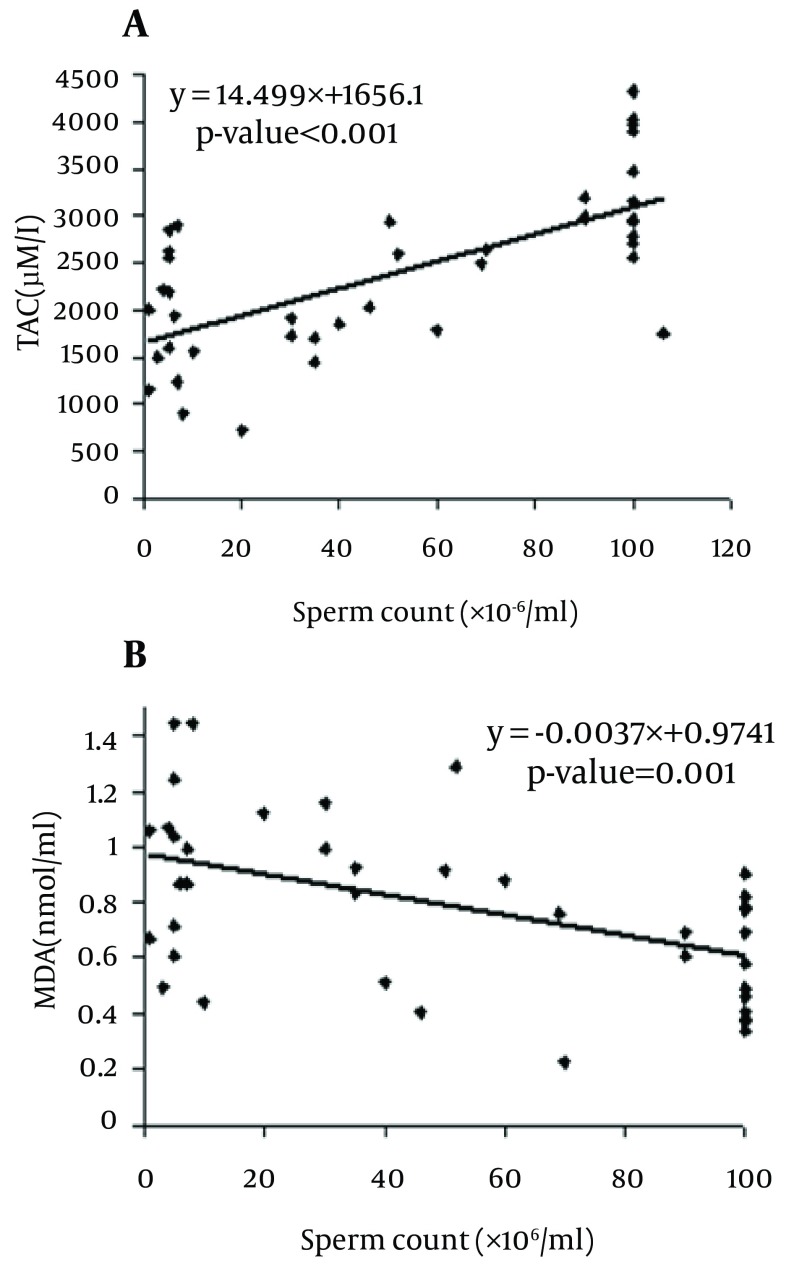
Correlations of Sperm Count (× 106/ml) With TAC A) MDA, B) Correlation is significant at P < 0.05 level (2-tailed).

MDA levels in asthenoteratospermic and oligoasthenoteratospermic were significantly higher than in healthy men (P = 0.049 and P = 0.001, respectively), but a significant difference between the patient groups was not observed. In contrast, the TAC in asthenoteratospermic and oligoasthenoteratospermic was significantly lower than in healthy men (P < 0.001, respectively), but a significant difference between the TAC in the patient groups was not seen. We also observed a negative correlation between MDA levels with the motility (P = 0.048), morphology (P = 0.001) and sperm count (P = 0.001) of spermatozoa, but a positive correlation between the TAC with motility (P < 0.001), morphology (P < 0.001) and sperm count (P < 0.001) in asthenoteratospermic and oligoasthenoteratospermic men. There was a negative correlation between MDA levels with the sperm count (× 106/ml) (P = 0.001) and a positive correlation between the TAC with the sperm count (× 106/ml) (P < 0.001) (Figure 2, 3 and 4).

## 5. Discussion

Oxidative stress (OS) as a result of an inappropriate balance between oxidants and antioxidants in the semen can lead to sperm damage, impairs the structure and function of spermatozoa and eventually male infertility (29, 30) OS results from Reactive Oxygen Species (ROS) or free radicals. In physiological conditions, ROS are required for crucial aspects of sperm function, in pathological conditions, excessive levels of ROS can negatively affect sperm quality (31). Sperm plasma membrane is particularly susceptible to lipid peroxidation by ROS due to the existence of high concentration of polyunsaturated fatty acids. Lipid peroxidation can lead to loss of membrane fluidity and integrity, as a result of this, reduces sperm-oocyte fusion. Furthermore, they can attack DNA by inducing base modifications, DNA strand breaks, DNA cross-links, and chromosomal rearrangements (32). Overproduction of ROS is associated with defective sperm function (33, 34). It is necessary a fine balance between ROS production and recycling, for successful spermatogenesis. There are some potential origins of ROS such as, seminal leukocytes with positive proxidase, immature, morphologically abnormal sperm, can be a cause of male infertility (35). Exposing the spermatozoa to ROS, causes DNA damage and lipid peroxidation (36). High lipid peroxidation may lead to reduce acrosomal reaction, fertilization (37) , and sperm oocyte fusion.

Malondialdehyde can be used as a marker of oxidative stress and a potential marker for predicting assisted reproductive techniques (ART) outcomes (38, 39). Several studies have shown that lipid peroxidation impacts the sperm concentration, motility, morphology and associated with poor sperm quality (40-42) Patel et al. observed (43) the negative correlation between the MDA levels and the normal sperm motility and morphology, so they suggested damaging effect of free radicals on sperm membrane integrity. Asbagh et al. (44) showed that there was significant association between semen MDA and abnormal sperm morphology, and decrease semen TAC and weak sperm motility. Gholinezhad and Hosseinzadeh Colagar6 showed a negative correlation between MDA levels and the motility and morphology of spermatozoa and a positive correlation between the TAC reflected by motility and morphology of spermatozoa in asthenoteratospermic smokers and non-smokers in comparison with a control group. In this study, we found Seminal TAC in asthenoteratospermic and oligoasthenoteratospermic men was significantly lower than in healthy (P < 0.001 respectively). Our results are in accordance with the study of Badade et al. (45), Koca et al. (46) and Khosrowbeygi et al. (47) Badade et al. (45) reported that TAC levels significantly lower in infertile men compared to fertile men. Khosrowbeeygi et al. (47) showed that TAC levels significantly lower in the asthenospermic, asthenoteratospermic and oligoasthenoteratospermic versus control group. Moreover, MDA levels in the control group were significantly lower than in asthenoteratospermic and oligoasthenoteratospermic patients (P = 0.049 and P = 0.001, respectively). A negative correlation between MDA levels with Sperm count, motility and normal morphology also a positive correlation in the TAC with Sperm count, motility and normal morphology between control group and asthenoteratospermic and oligoasthenoteratospermic men was observed. Our results of MDA are concurrent with Kobayashi et al. (48), Parineeta et al. (49) and Badade et al. (45) Kobayashi et al. (47) demonstrated high seminal MDA level in patients with oligoasthenoteratozoospermia. More over our results are in contrast with Suleiman et al. (50) They showed that MDA level in the seminal plasma was not correlated with the sperm concentration and motility.

Nabil et al. (51) observed MDA concentration in oligozoospermic and azoospermic men was significantly higher than normozoospermic while glutathione, ascorbic acid and total antioxidant status were significantly reduced in oligozoospermic and azoospermic men compared to normozoospermic group. Our study suggests, high ROS levels in semen lead to lipid peroxidation represented by MDA in semen, which may contribute to low motility, morphology and sperm count in the spermatozoa of asthenoteratospermic and oligoasthenoteratospermic patients. It is also important to determine the antioxidant status of semen as it is a major defense mechanism. The increase in malondialdehyde, and decrease in TAC levels in astheno- and oligoastheno-teratospermic men may have significant role in the etiology of sperm dysfunction. Positive correlation of TAC with sperm count, sperm motility and morphology, and negative correlation of sperm parameters with MDA indicate oxidative stress has a harmful effect in male infertility. Thus evaluation of seminal MDA and TAC could be beneficial diagnostic tool for defining sperm fertilization potential. These parameters could help in distinction and treatment of male infertility especially in idiopathic cases.
